# Curbing the menace of antimicrobial resistance in developing countries

**DOI:** 10.1186/1477-7517-6-31

**Published:** 2009-11-19

**Authors:** Chidi Victor Nweneka, Ndey Tapha-Sosseh, Anibal Sosa

**Affiliations:** 1 Medical Research Council Laboratories, Keneba Field Station, P.O. Box 273, Banjul, The Gambia; 2 Alliance for the Prudent Use of Antibiotics, The Gambia, P.O. Box 3416, Serekunda, The Gambia; 3 Alliance for the Prudent Use of Antibiotics (APUA), 75 Kneeland Street, Boston, MA 02111, USA

## Abstract

Several reports suggest that antimicrobial resistance is an increasing global problem; but like most pandemics, the greatest toll is in the less developed countries. The dismally low rate of discovery of antimicrobials compared to the rate of development of antimicrobial resistance places humanity on a very dangerous precipice. Since antimicrobial resistance is part of an organism's natural survival instinct, total eradication might be unachievable; however, it can be reduced to a level that it no longer poses a threat to humanity. While inappropriate antimicrobial consumption contributes to the development of antimicrobial resistance, other complex political, social, economic and biomedical factors are equally important. Tackling the menace therefore should go beyond the conventional sensitization of members of the public and occasional press releases to include a multi-sectoral intervention involving the formation of various alliances and partnerships. Involving civil society organisations like the media could greatly enhance the success of the interventions

## Introduction

It is difficult to determine the worldwide prevalence of antimicrobial resistance (AMR); but several reports suggest that it is an increasing problem of phenomenal proportions, affecting both rich and poor countries [1-8]. In 2007, the prevalence of Methicillin-resistant *Staphylococcus aureus *(MRSA) ranged from 27.4 to 62.4% and Penicillin-nonsusceptible *Streptococcus pneumoniae *from 23.3% to 54.5% in the different census regions of the United States[1]. In the UK, enterobacteriacea resistance to cephalosporins is on the increase[2], as is the prevalence of MRSA[3] in hospital and community settings. The recent report of the European Antimicrobial Resistance Surveillance System showed a rising prevalence of resistance among the seven bacterial species (*Streptococcus pneumoniae, Staphylococcus aureus, Escherichia coli, Enterococcus faecalis, Enterococcus faecium, Klebsiella pneumoniae and Pseudomonas aeruginosa*) that serve as indicators for the development of antimicrobial resistance in Europe to many of the mainline antibiotics[4]. In India, up to 80% of *S. aureus *strains are resistant to penicillin and ampicillin[5]. Of 3362 pneumococcal isolates collected from 69 centres in 25 countries in the PROTEKT (Prospective Resistant Organism Tracking and Epidemiology for the Ketolide Telithromycin) study between 1999 and 2000, resistance to Penicillin G was 53.4% in Asia (overall prevalence), France 46.2%, Spain 42.1% and North Korea 71.5%; resistance to erythromycin varied from 4.7% in Sweden to 87.6% in South Korea; while resistance to fluoroquinolones in Hong Kong was 14.3%[6]. And in South Africa, macrolide resistance and penicillin non-susceptibility were 54% and 74% respectively[7]. Chloroquine is almost useless as an antimalarial in most malaria endemic countries, while MDR-TB and XDR-TB are now assuming frightening proportions[9]. While AMR is a growing global problem, like most epidemics, the greatest toll is usually in the less developed countries. Unfortunately, the rate at which antimicrobial resistance is developing far outstrips the rate at which new antimicrobials are being discovered, placing humanity on a very dangerous precipice.

AMR, as an attempt by the organisms to survive, is a natural phenomenon[5]. It is a reality that will remain with us; although it can be slowed, it can not be completely eradicated. The question therefore is how the rate of development of AMR can be slowed down to a level that maintains the usefulness of the antimicrobials, insuring that humanity is not annihilated.

## Discussion

### Curbing antimicrobial resistance

Finland [10] had proposed that the dominant factor in the emergence and spread of antibiotic-resistant bacterial pathogens is the intensive use of antibiotic agents; suggesting a strong influence of behavioural factors in the development of AMR, both from prescribers and patients. However, the correlation between the intensity of antimicrobial use and resistance has not been consistent [11-15]. Thus, while antimicrobial consumption facilitates the development of AMR, other complex factors need serious considerations.

Answering the question of how AMR could be slowed to 'acceptable' limits is not straightforward as the development of AMR results from a complex interplay of several biomedical, behavioural, socio-economic and political factors [16,17]. Accordingly, several solutions have been proposed including education of health care and allied professionals and the general public, basic research and surveillance mechanisms at various levels [18]; and regulation of over the counter drugs [5]. Each of these suggested solutions have their merits but will achieve limited success in developing countries unless some basic issues are addressed.

### Basic issues

Poverty is a major factor in the development of AMR in developing countries [19]. Poverty encourages the patronage of quack health care practitioners and medicine vendors who dispense sub-standard, counterfeit or expired drugs or sub-therapeutic doses of antimicrobials. Poverty also encourages self-medication due to inability to access health care services. Furthermore, paucity of qualified health care workers (HCWs) in many developing countries, poorly maintained and dilapidated health care facilities and poor access to health care further encourage quackery. In places where health care facilities are available, the cost prevents access to such services. Ignorance and illiteracy also contributes to under-dosing and under-treatment by many patients who are quick to discontinue their treatment once they feel better. Furthermore, anecdotal evidence shows that many traditional healers in parts of Africa add antibiotics to some of the concoctions they administer to their clients. The effect of such antibiotic-laced concoctions would be to accumulate in the system of the recipients at sub-therapeutic doses, increasing the chances of resistance developing. Corruption which is a global pandemic encourages the importation of sub-standard drugs and the misappropriation of funds meant for the improvement of health care services [20]. And directly related to this is the exploitation of the vulnerabilities of third world countries by the better privileged advanced countries.

The solution to these 'basic' problems highlighted above would appear straightforward: Enact laws on antimicrobial use, enforce existing laws on antimicrobial use, regulate antimicrobial agents as special class of drugs, provide further education of health care providers and the general public on anti-microbial use and antimicrobial resistance, provide improved access to health care facilities, train more HCWs, tackle poverty and ensure better enforcement of international laws governing drug production and export. Other approaches would include the retention of prescriptions by the pharmacies, training drug-sellers, and regulating the kind of drugs that the drug-sellers can sell and dispense.

### Multi-sectoral intervention needed

Unfortunately, these are no easy solutions. Several social, political and economic factors hinder their implementation. One way to get around the hurdles in the way of implementing these solutions is to adopt a multi-sectoral approach involving the formation of various alliances: Grassroots organizations such as the Alliance for the Prudent use of Antibiotics (APUA), professional - professional partnerships, professional - civil society organisations (CSOs) partnerships, private - government partnerships, and private-private partnerships. Effective involvement of CSOs could enhance the success of health care intervention programmes. One contemporary example is the vaginal microbicide, the visibility of which was greatly facilitated by the strong advocacy mounted by several civil society organisations over the last 20 years. Biomedical solutions alone will not curb the menace of AMR, nor would epileptic press releases and ambiguous community education efforts. Efforts to educate the public and other stakeholders will not be effective without the active involvement of CSOs. Sustained high profile advocacy and sensitizations are needed to create a massive awareness of the danger posed by AMR, and to influence behavioural change. This can be achieved by effective engagement of sectors like the CSOs. The CSOs are better experienced in advocacy; communicate better with the grassroots with whom they enjoy a close association; and they have better negotiating skills with governments and other relevant agencies, and in resource mobilization for research. Also, CSOs are likely to devote more time to advocacy and community and stakeholder sensitization than professional organisations.

### The media as a potential partner

One sector that could play a very important role in curbing the menace of AMR is the media. The trained media professionals can help to adequately convey information not only about the effects of antibiotic misuse to the individual, but also about the way in which it affects the wider community. A story about antibiotic misuse, for instance, could seem removed from peoples' daily lives unless the reporter explains the potential health threat in human terms. People will care about an issue provided they are given reasons to care. However, to discharge this task effectively, the relevant government and professional bodies need to assent to a commitment to transparency. There is no excuse to hide information from the media, the public, or from other governments and international agencies that are seeking to curb the menace of antibiotic misuse. But, a commitment to transparency on its own is insufficient. Equally important is the need to ensure that those in the front-line of public health communication - namely science and health journalists - have adequate tools and skills to perform their task, for example to detect when a commitment to transparency is not being observed.

It will be in everyone's interest for governments and health-related organizations to recognise and acknowledge that responsible health reporting can play a significant role in limiting antibiotic misuse. It is clear that effectively communicating accurate information about the use/misuse of antibiotic will be essential to efforts to contain its misuse. There are a number of practical reasons for this. It is important, for instance, for the public to know that non-continuation of a dose of an antibiotic prescription can lead to resistance to it, a practice that is very common in many developing countries. Most people, once on the road to recovery discard medicines without any knowledge of the grave danger to which they expose themselves, their families and communities. The media will be the best channel of such information to the public. With widespread distrust of many public institutions in developing countries, partnering with media organizations to develop and probably assist in running health programmes with an emphasis on antibiotic use/misuse could prove a worthwhile investment, the intended outcome being to reach and change the behaviour of as many people as possible in order to curb the menace of antibiotic misuse. Directly related to this is effective use of Behaviour Change Communication and Information Education and Communication materials, which can be done in partnership with media outfits and other NGOs interested in health and development issues. Local drama groups and customary messengers like the *kanyelengs *in The Gambia could also be utilized effectively to disseminate the message of AMR as their importance in local and rural communities is deeply rooted. Such partnerships as outlined here usually take a comprehensive communications approach combining targeted public messages, the integration of messages into popular shows and the extensive use of news media.

The choice of media - whether print or electronic - through which to disseminate the information is also important. The television provides a dual route for conveying a message - through the spoken word and through images. Through some creativity better effects could be achieved through the television. The influence of the radio could be grossly underestimated and occasionally overlooked by even the most experienced media strategists. Radio is often described as the "captive electronic medium" (WORKING WITH THE MEDIA, http://legacy.kctcs.edu/newspublications/stylebook/mediatypes.htm) because it reaches people while they are doing other things - in their cars, on the way to and from work, in their homes and offices, at the farms. The print media also have their places in the fight against antimicrobial resistance.

### Traditional forms of Communication

The potential of folk drama and traditional forms of communication in folk theatre, folksongs, narrative forms and religious discourses as an important channel of communication is often overlooked. Developed messages can be conveyed to these groups for further dissemination to local communities. Folk communication easily escapes a lot of the problems encountered by the mass media in the integration process as they are already an integral part of the community. Thus if the battle to curb the menace of antibiotic misuse is to be won, media practitioners (both Western and traditional) at various levels must be brought on board and better strategies developed to maximize the use of the different types of media outlets.

## Conclusion

In conclusion, AMR is real and ravaging all countries. While laboratory based and clinical studies are important in elucidating the problem, they are not enough. A multisectoral, multi-disciplinary approach involving CSOs and the media offers the best option for slowing the rate of development of AMR.

## List of abbreviations

AMR: antimicrobial resistance; APUA: Alliance for the Prudent Use of Antibiotics; CSOs: civil society organisations; HCWs: health care workers; MRSA: methicillin resistant staphylococcal aureus; UK: United Kingdom.

## Competing interests

The authors declare that they have no competing interests.

## Authors' contributions

All the authors generated the ideas; CVN was the lead writer but all the other authors contributed and reviewed the final manuscript.
